# Gut Microbial Characterization of Melon-Headed Whales (*Peponocephala electra*) Stranded in China

**DOI:** 10.3390/microorganisms10030572

**Published:** 2022-03-06

**Authors:** Shijie Bai, Peijun Zhang, Xianfeng Zhang, Zixin Yang, Songhai Li

**Affiliations:** 1 Institute of Deep-Sea Science and Engineering, Chinese Academy of Sciences, Sanya 572000, China; baishijie@idsse.ac.cn (S.B.); yangzx@idsse.ac.cn (Z.Y.); 2 Institute of Hydrobiology, Chinese Academy of Sciences, Wuhan 430072, China; zhangx@ihb.ac.cn

**Keywords:** melon-headed whale, gut, microbial communities, aquatic mammal

## Abstract

Although gut microbes are regarded as a significant component of many mammals and play a very important role, there is a paucity of knowledge around marine mammal gut microbes, which may be due to sampling difficulties. Moreover, to date, there are very few, if any, reports on the gut microbes of melon-headed whales. In this study, we opportunistically collected fecal samples from eight stranded melon-headed whales (*Peponocephala electra*) in China. Using high-throughput sequencing technology of partial 16S rRNA gene sequences, we demonstrate that the main taxa of melon-headed whale gut microbes are Firmicutes, Fusobacteriota, Bacteroidota, and Proteobacteria (Gamma) at the phylum taxonomic level, and *Cetobacterium*, *Bacteroides*, *Clostridium sensu stricto*, and *Enterococcus* at the genus taxonomic level. Meanwhile, molecular ecological network analysis (MENA) shows that two modules (a set of nodes that have strong interactions) constitute the gut microbial community network of melon-headed whales. Module 1 is mainly composed of *Bacteroides*, while Module 2 comprises *Cetobacterium* and *Enterococcus*, and the network keystone genera are *Corynebacterium*, *Alcaligenes*, *Acinetobacter*, and *Flavobacterium*. Furthermore, by predicting the functions of the gut microbial community through PICRUSt2, we found that although there are differences in the composition of the gut microbial community in different individuals, the predicted functional profiles are similar. Our study gives a preliminary inside look into the composition of the gut microbiota of stranded melon-headed whales.

## 1. Introduction

The melon-headed whale (*Peponocephala electra*) is a member of the subfamily Globicephalinae, where it is most closely related to the larger pilot whales (*Globicephala melas *and* G. macrorhynchus*), and it is also not a well-known species [1]. This whale is mostly dark gray in color, with a faint dark gray cloak on its back and a narrow head that slopes downward below a tall sickle-shaped dorsal fin. This species is difficult to distinguish at sea from the pygmy killer whale (*Feresa attenuata*). However, in stranded specimens, the melon-headed whale can be identified from all other pygmy killer whales by its high tooth count, as the melon-headed whale has ~25 teeth per row, while the pygmy killer whale has only about ~15 teeth per row [2]. Melon-headed whales are found worldwide in tropical and warm–temperate waters [3]. They mainly feed on fish, squid, cuttlefish, and shrimp, foraging from the littoral zone down to the bathypelagic zone [2,4,5].

Microbes are exceedingly abundant and varied in the gut of mammals [6]. Interactions between microbes and their host are necessary for the regulation of health, survival, and physiological functions of the host [7,8,9]. The majority of microbes reside in the gut, and their associated phenotypes shape the immune system of the host and contribute to nutrient uptake and defense against infectious diseases [10,11]. Therefore, revealing the mammalian gut microbiota is essential to fully understand the physiology and health status of mammals themselves. To date, most studies have focused on human gut microbiota, and information on the gut microbial composition of other mammals, especially cetaceans, although there are some reports, remains relatively scarce due to sampling limitations.

According to previous reports, gut samples from cetaceans are mainly obtained from three approaches: (1) feces in the wild just post-defecation. For example, Sanders et al. [12] investigated the microbial diversity and function of gut microbiomes in baleen whales feces and found them harbored unique gut microbiomes whereas still kept a functional capacity similar to that of both carnivores and herbivores; (2) fecal samples from human cared animals, such as studies on belugas (*Delphinapterus leucas*), Pacific white-sided dolphins (*Lagenorhynchus obliquidens*) and common bottlenose dolphins (*Tursiops truncatus*) [13,14], and Yangtze finless porpoises (*Neophocaena phocaenoides asiaeorientalis*) [15]; and (3) from dead, stranded animals. A few of studies sequenced along the gastrointestinal tracts of stranded cetaceans to investigate the distribution of microorganisms in different gut regions [16,17,18,19].

In this study, we opportunistically collected fecal samples from eight melon-headed whales stranded in China. Through investigating this infrequently known cetacean species, we aim to address the gut microbial compositions and diversity and gut microbial community network and predict the potential function of gut microbes in melon-headed whales.

## 2. Materials and Methods

### 2.1. Sample Collection

A rare mass stranding of 12 melon-headed whales happened on 6 July 2021, Tumen Port, Linhai, Taizhou City, Zhejiang Province, China. In this group of melon-headed whales, three individuals were found dead, two were released back immediately during the rescue course, and the remaining seven individuals were temporarily kept for recovery and released back to the wild the next day. We thus collected seven fecal samples from the recovering melon-headed whales before their release. 

Another melon-headed whale stranding case happened on 25 May 2021, in Houan Town, Wanning City, Hainan Province, China. The animal was rescued and kept in Fuli Oceanarium (Lingshui, Hainan Province, China) for recovery. We collected one fecal sample from this animal on June 10 during its recovery time, before its death on 20 June 2021. 

All fecal samples were harvested by veterinarians using anal swabs, with a diameter of 12 mm, which were inserted 10–15 cm into the rectum. All fecal samples were collected when animals were lifted out of water, and frozen at −20 °C until DNA extraction. Detailed information of these sampling animals is shown in Table S1.

### 2.2. DNA Extraction and Sequencing

The DNA of all fecal samples and three extraction blank control samples were extracted using MoBio PowerSoil extraction kits (Mo Bio Laboratories, Carlsbad, CA, USA) in accordance with the manufacturer’s instructions. The extracted DNA was quantified using a Qubit fluorometer (Invitrogen Inc. Manufacturer: Life Technologies Holdings Pte Ltd., Singapore) and primer pair 515f Modified and 806r Modified were used to amplify the V4 region of the 16S rRNA gene [20]. The PCR amplification was performed under the following conditions: denaturation at 95 °C for 3 min, followed by 27 cycles at 95 °C for 30 s, 55 °C for 30 s, and 72 °C for 45 s, and a final extension at 72 °C for 10 min. PCR amplification results in triplicate were combined after purification with a TaKaRa purification kit (TaKaRa, Kusatsu, Japan). PCR products were prepared for library construction using the TruSeq DNA sample preparation kit (Illumina, San Diego, CA, USA) in accordance with the manufacturer’s instructions. The libraries were sequenced at MajorBio Co. Ltd. (Shanghai, China) using the HiSeq platform (Illumina, San Diego, CA, USA) with reads of 250 bp at the paired end [13].

### 2.3. Microbial Community Analysis

After sequencing and obtaining the raw data, barcodes were removed as well as forward and reverse primers (one mismatch each was allowed) to obtain clean data. The FLASH program version 1.2.8 [21] was used to obtain paired-end of sufficient length with at least a 30 bp overlap combined into full-length sequences, and the average fragment length was 253 bp. The high-quality sequences without Ns contained were recruited using the Btrim program (version 0.2.0), and the sequences of 245 bp to 260 bp were used for the next analysis [22]. UNOISE3 was applied to generate amplicon sequence variants (ASVs) with default settings [23]. A representative sequence from each ASV was selected for taxonomic annotation via comparison with the SILVA 132 database [24], which includes bacterial, archaeal, and eukaryotic sequences; the Chloroplast and mitochondrial reads were excluded. To take into count the different sequencing depths, ASVs were randomly resampled to normalize the reads for each sample. The diversity of the microbial communities from the fecal samples of different individuals was determined via statistical analysis of the α-diversity indices, such as the Shannon, Inverse Simpson, Chao1 indices [25], and observed richness. R language [26] and the Mothur program [27] were used to calculate these α-diversity indices. 

Molecular ecological network analysis (MENA) was used to perform the structure of microbial community networks [28,29]. Only the ASVs that appeared in more than four of the eight fecal samples of melon-headed whales were included in the network analysis. Correlations were calculated using Spearman’s coefficient and a random matrix theory (RMT)-based approach was employed to delimit the microbial network interactions between samples. The keystone taxa were allocated according to the within-module connectivity (Zi) and among-module connectivity (Pi) according to a previously used method [28]. Nodes (ASVs) can be divided into four categories: (1) peripherals, which includes the nodes with Zi ≤ 2.5 and Pi ≤ 0.62, indicating nodes interconnected by a few links within the modules; (2) connectors, which includes the nodes with Zi ≤ 2.5 and Pi > 0.62, indicating nodes linking to various modules; (3) module hubs, which includes the nodes with Zi > 2.5 and Pi ≤ 0.62, indicating nodes within the modules are highly connected; and (4) network hubs, which includes the nodes with Zi > 2.5 and Pi > 0.62, indicating nodes highly connected among modules. The Phylogenetic Investigation of Communities by Reconstruction of Unobserved States (PICRUSt2) was used to predict microbial community function based on the MetaCyc database [30,31]. The raw sequencing reads of all samples were deposited to the NCBI database (http://www.ncbi.nlm.nih.gov/, accessed on 29 January 2022) under BioProject accession number: PRJNA801934.

## 3. Results

### 3.1. Sequencing Statistics and Microbial Diversity

**Figure 1:**
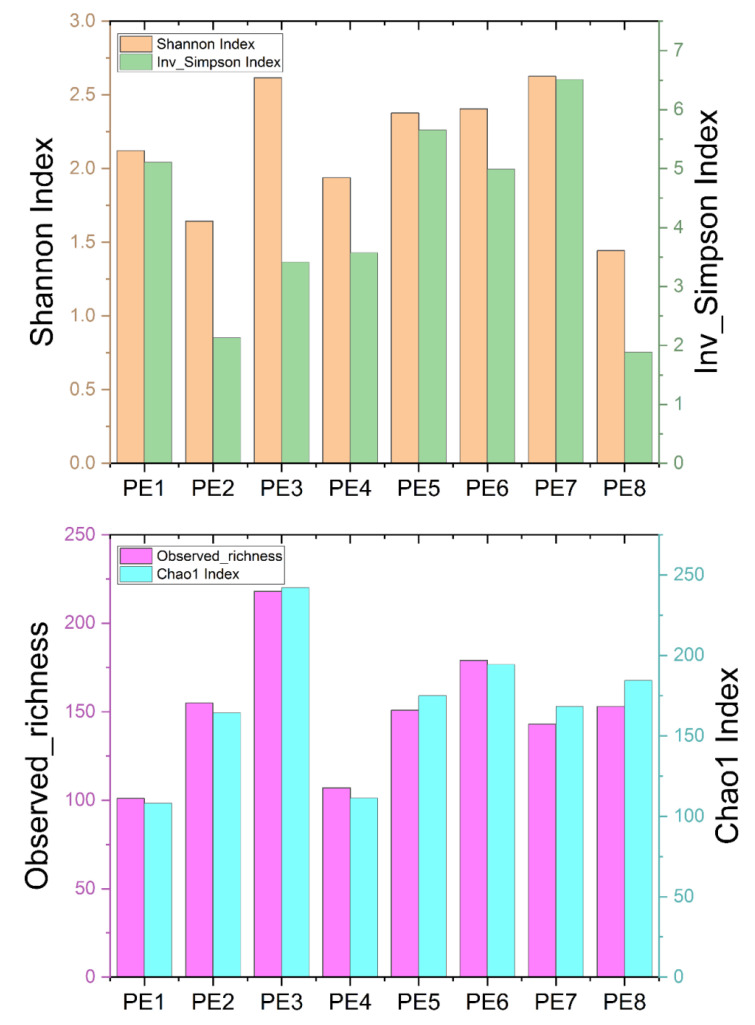
Four α-diversity indices—Shannon index, Inverse Simpson index, observed richness, and Chao1 index—of the eight fecal specimens from eight stranded melon-headed whales (PE1-8). The results are based on the ASV datasets.

Originally, a total of 642,263 sequences were obtained from 8 fecal samples of 8 stranded melon-headed whales (assigned as PE1 to PE8, Table S1) after quality assessment. To obtain more accurate α-diversity results to analyze microbial diversity, composition, and structure, we rarefied the sequences of each sample to 34,224. The α-diversities of microbial communities from the gut of eight melon-headed whales were calculated. The results showed PE8 and PE2 had lower Shannon and Inverse Simpson indices, while PE1 and PE4 had lower Chao1 indices and observed richness (Figure 1). 

**Figure 2:**
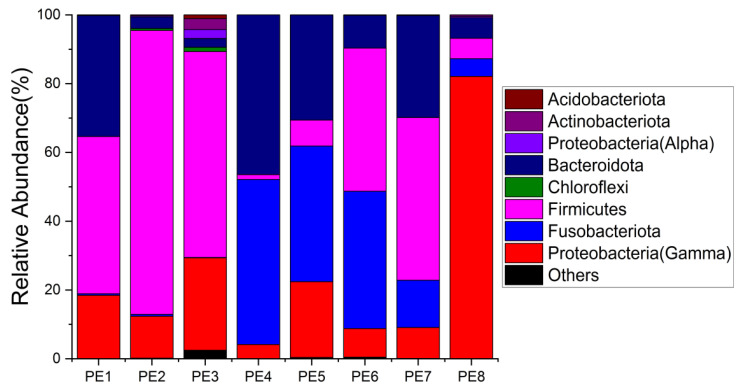
Gut microbial community members of eight stranded melon-headed whales (PE1-8) at the phylum level.

**Figure 3:**
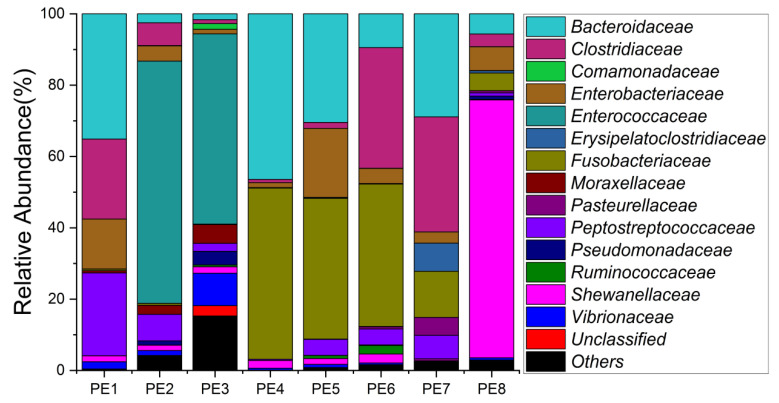
Gut microbial community members of eight stranded melon-headed whales (PE1-8) at the family level.

**Figure 4:**
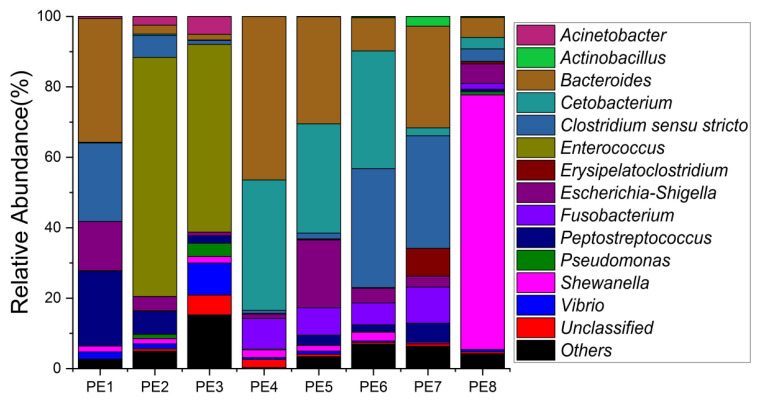
Gut microbial community members of eight stranded melon-headed whales (PE1-8) at the genus level.

The relative abundance of gut microbes was apparent at the phylum, family, and genus levels, with a similarity of 97% for ASV taxonomy, and provided detailed relative abundance information on gut microbial community composition (Figure 2, Figure 3 and Figure 4). Furthermore, we also provided the datasets of ASV table and the information of classification (Table S2). Firmicutes, Fusobacteriota, and Bacteroidota were the dominant bacterial lineages in the fecal samples of melon-headed whales, while the majority of the fecal samples from the PE8 in this study were dominated by Proteobacteria (Gamma), accounting for 82%. At the family taxonomic level, Fusobacteriaceae, Enterococcaceae, and Bacteroidaceae, which are affiliated with Fusobacteriales, Lactobacillales, and Bacteroidales, respectively, were the dominant bacterial lineages in the fecal samples of PE1 to PE7. However, the respective compositions of different fecal samples were slightly different; for instance, the fecal sample of PE8 was dominated by Shewanellaceae (Enterobacterales, 72%). Furthermore, at the genus taxonomic level, the gut microbial communities of melon-headed whales were mainly composed of Cetobacterium, Bacteroides, Clostridium sensu stricto, and Enterococcus. Nevertheless, the distribution of these dominant bacterial lineages in different fecal samples is different. For instance, Cetobacterium was dominant in the fecal samples of PE4, PE5, and PE6; Bacteroides was dominant in the samples of PE1, PE4, and PE7; and Clostridium sensu stricto was dominant in the samples of PE1, PE6, and PE7. The fecal samples of PE2 and PE3 were dominated by Enterococcus, which accounted for 68% and 53%, respectively. Only one ASV was annotated with Shewanella, and this ASV was annotated at the level of species as Shewanella algae. This bacterium was distributed in all fecal samples, but in the sample of PE8, Shewanella algae was the overwhelmingly dominant bacterium, accounting for 72% (Figure 4). 

### 3.2. Co-Occurrence Network and Functional Profile of Gut Microbial Communities

**Figure 5:**
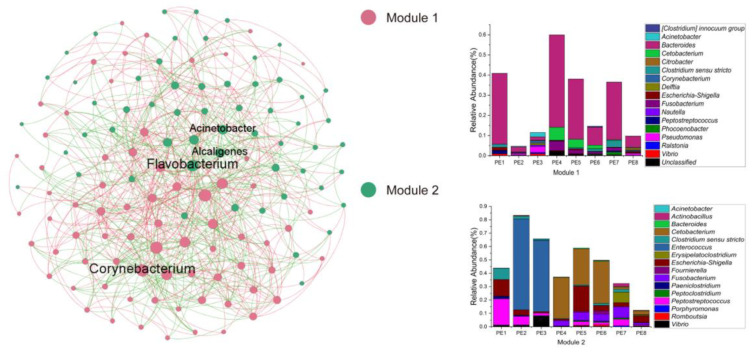
Co-occurrence networks of gut microbial communities. Stacked bar chart shows relative abundance of ASVs in Modules 1 and 2; a Module is a set of nodes that have strong interactions; these samples were collected from eight stranded melon-headed whales (PE1-8).

In order to reveal the gut microbial community interactions of melon-headed whales, the network was constructed through the MENA approach. The nodes and links of this network were 128 and 676, respectively. The average clustering coefficient (avgCC) was 0.337, and the average path distance (GD) was 2.513. This network formed a total of two modules (a set of nodes that have strong interactions): module one was mainly composed of *Bacteroides*, while module two was mainly composed of *Cetobacterium* and *Enterococcus*. Moreover, the keystone taxa belonged to module hubs, composed of those ASVs with Zi > 2.5, Pi ≤ 0.62, in the microbial network of melon-headed whales; the keystone genera were *Acinetobacter*, *Alcaligenes*, *Corynebacterium*, and *Flavobacterium* (Figure 5). 

**Figure 6:**
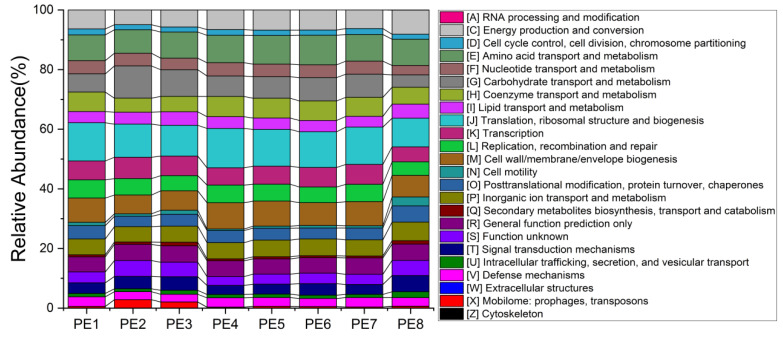
Functional profiles of gut microbial communities predicted by PICRUSt2; these samples were collected from eight stranded melon-headed whales (PE1-8).

To better understand the potential functions of melon-headed whale gut bacteria, we explored the functional features of microbial communities using the newly updated PICRUSt2 software. No obvious functional difference was found between individuals. The main functions involved in the gut microbes of stranded melon-headed whales include the following: RNA processing and modification; energy production and conversion; cell cycle control, cell division, chromosome partitioning; amino acid transport and metabolism; nucleotide transport and metabolism; carbohydrate transport and metabolism; coenzyme transport and metabolism; lipid transport and metabolism; translation, ribosomal structure, and biogenesis; transcription; replication, recombination, and repair; cell wall/membrane/envelope biogenesis; cell motility; post-translational modification, protein turnover, chaperones; inorganic ion transport and metabolism; secondary metabolites biosynthesis, transport, and catabolism; signal transduction mechanisms; intracellular trafficking, secretion, and vesicular transport, and defense mechanisms (Figure 6). The detailed results of PICRUSt2 were provided in Table S3.

## 4. Discussion

Due to the difficulty of sample collection, studies on cetacean gut microbes are usually from animals in zoos and oceanariums (e.g., [13,14,32]), or stranded cetaceans (e.g., [16,17,19]). To date, there are very few, if any, reports on the gut microbial communities of melon-headed whales. In this study, we obtained eight fecal samples from eight different stranded melon-headed whales. Through 16S rRNA gene sequencing, we revealed that members of *Cetobacterium*, *Bacteroides*, *Clostridium sensu stricto*, and *Enterococcus* constituted the vast majority of the gut microorganisms in melon-headed whales. We also found the distribution of gut microorganisms in different individuals was different; in spite of this, the functional profiles between individuals were similar. Thus, we propose that a functional-driven strategy may play an important role in the composition of the gut microbial community in melon-headed whales, rather than a species-driven strategy. However, further studies are warranted.

We also want to mention that PE8 in our study was not healthy, and was treated with antibiotics, i.e., penicillins and cephalosporin, for two weeks under human care before sample collection. Antibiotic treatment had a potential to affect the composition of gut microbial communities in PE8. A necropsy of PE8 showed it suffered from lung lesion, which might be the reason of its death. When we document the composition of gut microbial communities in melon-headed whales in our study, we always carefully consider the situation of PE8 first, and then make a cautious conclusion.

The genera *Cetobacterium*, from the phylum Fusobacteria, can be found in the gut of many cetacean species, such as short-finned pilot whales [16], toothed whales [12], and southern right whales *Eubalaena australis* [33]. Polysaccharides comprise the most abundant type of biopolymers, and therefore, the most abundant source of biological food. Carbohydrate fermentation by *Bacteroides* and other intestinal bacteria produces large amounts of volatile fatty acids, which are absorbed through the large intestine and utilized by the host as an energy source, providing a large portion of the host’s daily energy needs [34]. Although most *Bacteroides* are symbiotic in the intestine, several species can also cause infections, including *Bacteroides fragilis, Bacteroides distasonis, Bacteroides ovatus, Bacteroides thetaiotaomicron, Bacteroides vulgatus*, and *Bacteroides uniformis*, with significant morbidity and mortality [35]. The genera *Clostridium sensu stricto* are other common microorganisms in the gut of cetaceans, for example, beluga whales *Delphinapterus leucas*, Pacific white-sided dolphins *Lagenorhynchus obliquidens*, common bottlenose dolphins *Tursiops truncatus*, and short-finned pilot whales [13,16]. *Clostridium* is one of the most common genera of cetacean gut microorganisms, while some studies suggest that members of *Clostridium* have low virulence and can pose a potential threat to unhealthy cetaceans [33,36]. The members of *Enterococcus* can also be found in the gut of some cetaceans, such as pygmy sperm whales *Kogia breviceps*, Pacific white-sided dolphins, and common bottlenose dolphins [13,16]. 

We detected an overwhelming dominance of *Shewanella algae* in PE8. However, the group of *Shewanella algae* was not found to be particularly common in the gut of cetaceans in this or in previous studies; we detected this bacterial linage in the gut of short-finned pilot whales[16] and melon-headed whales. Furthermore, the ASVs were all annotated as *Shewanella algae*. *Shewanella algae* is ubiquitous in the marine environments and has been identified as conditionally pathogenic bacteria that can cause serious infections, primarily associated with exposure to seawater and ingestion of raw seafood, and this group of bacteria can exhibited b-hemolytic activity, strong biofilm-adherence capabilities, and multiple antibiotic resistances [37,38,39,40,41,42]. We think that *Shewanella algae* should not be a dominant group (though it can be present) in melon-headed whales or short-finned pilot whales [16]; indeed, the overwhelming dominance of *Shewanella algae* in the gut of PE8 might have been a potential trigger of its death (Figure 3). A necropsy of PE8 showed that it likely died of lung lesion.

Functional profiles are characteristics that influence the adaptability of microbial communities under specific environmental conditions. However, because of the continuous exchange and transfer of horizontal genes between microorganisms and adaptive evolution, functional characteristics of microbial communities can be delinked from their taxonomic relevance [43]. In the present study, although there were differences in the microbial community structures between different samples, their predicted functional profiles were similar. The recently developed approach of molecular ecological networks can reveal the interrelations within a microbial community. We found two modules in the gut microbial community network of the eight stranded melon-headed whales. The microbial communities of Module 1 were dominated by *Bacteroides*, and the keystone genus was *Corynebacterium*. *Cetobacterium* and *Enterococcus* were the dominant bacterial lineages in Module 2, and *Alcaligenes*, *Acinetobacter*, and *Flavobacterium* were the keystone genera. The genus *Corynebacterium* represents a group of Gram-positive, rod-shaped, and typically club-shaped bacterial cells [44]. Some species of *Corynebacterium* are well-known pathogens of mammals and may occasionally cause infections, while some other species are normal microorganisms of microbial communities where it belongs [44]. In this study, ASV 56 was the keystone genus of Module 1 and could be annotated to the genus level. The keystone ASVs of Module 2, *Alcaligenes* and *Acinetobacter*, could also be annotated to the genus level, while another keystone ASV of Module 2, annotated as *Flavobacterium jumunjinense*, was isolated from lagoon water in Korea [45]. The genus *Alcaligenes* consists of motile Gram-negative rod-shaped bacteria that are chemoorganotrophic microbes. The members of *Alcaligenes* are common in water, soil, vertebrate intestinal tracts, and in clinical samples as a result of opportunistic infection [46]. Some *Alcaligenes* strains are able to be isolated from some contaminated environmental samples; therefore, they may show potential in the development of biodegradation processes or as biosensors. Moreover, some species of *Alcaligenes* are used in the food and healthcare industries, while some enzymes and polysaccharides produced by *Alcaligenes* have been used in the cosmetic industry and as food additives, showing potential for the treatment of certain immune diseases [46]. *Acinetobacter* spp. are Gram-negative coccobacilli; they are ubiquitous in the environment and are considered to be nonpathogenic to healthy individuals [47]. Although we detected both groups of bacteria (*Alcaligenes* and *Acinetobacter*) in the gut of melon-headed whales, their relative abundance was very low, and their roles are still unclear.

## 5. Conclusions

It is important to reveal the gut microbial communities of specific cetacean species, especially some poorly understood ones. In our study, the composition, functional profile, and interactions of gut microbial communities of eight stranded melon-headed whales were systematically studied. We conclude that the microbial community composition mainly consists of *Cetobacterium*, *Bacteroides*, *Clostridium sensu stricto*, and *Enterococcus.* Two modules constitute the network of the gut microbes of melon-headed whales; *Bacteroides* was the main microbial taxon in Module 1, while Module 2 mainly comprised *Cetobacterium* and *Enterococcus*. Moreover, based on network analysis, the keystone taxa (module hubs) were assigned to *Corynebacterium*, *Alcaligenes*, *Acinetobacter*, and *Flavobacterium*. Our study gives a preliminary inside look into the composition of the gut microbiota of stranded melon-headed whales. Furthermore, we also want to mention that we have very limited microbial information in melon-headed whales, as only one group of whales was studied. This may strongly affect the informational value of the obtained data. All whales may have had an exchange of the microbiota and may have been affected by the same environmental conditions. Other studies of whale feces microbiota studied samples collected from whales at different locations and different time points should be further conducted. In addition, metagenomics, transcriptomics, and proteomics should be used to better understand the functional information of the gut microbes in melon-headed whales.

## Data Availability

The raw sequencing reads of all samples were deposited to the NCBI database (http://www.ncbi.nlm.nih.gov/, accessed on 29 January 2022)) under BioProject accession number: PRJNA801934.

## Supplementary Materials

The following supporting information can be downloaded at: https://www.mdpi.com/article/10.3390/microorganisms10030572/s1, Table S1: Sampling information of the eight melon-headed whales; Table S2: ASV datasets and the classification information; Table S3: The detailed functional results predicted by PICRUSt2.
