# Supplementary material for: Gut Microbial Characterization of Melon-Headed Whales (Peponocephala electra) Stranded in China
Source: Microorganisms. 2022 Mar 6;10(3):572. doi: 10.3390/microorganisms10030572 (PMC8950688; doi:10.3390/microorganisms10030572)
Supplement: Supplementary file 1 [file microorganisms-10-00572-s001.zip › microorganisms-1599046-S/Table_S1.pdf]

Table S1. Sampling information of the 8 melon-headed whales

Sample ID	Stranding date	Sampling date	Species name	Stranding city	Sex	Age
**PE1**	July 6th	July 7th	Melon-headed whale	Taizhou, China	F	Adult
**PE2**	July 6th	July 7th	Melon-headed whale	Taizhou, China	F	Adult
**PE3**	July 6th	July 7th	Melon-headed whale	Taizhou, China	M	Adult
**PE4**	July 6th	July 7th	Melon-headed whale	Taizhou, China	M	Adult
**PE5**	July 6th	July 7th	Melon-headed whale	Taizhou, China	M	Adult
**PE6**	July 6th	July 7th	Melon-headed whale	Taizhou, China	F	Adult
**PE7**	July 6th	July 7th	Melon-headed whale	Taizhou, China	M	Adult
**PE8**	May 25th	June 10th	Melon-headed whale	Wanning, China	F	Subadult
